# Changes in cytokine and chemokine profiles in mouse serum and brain, and in human neural cells, upon tick-borne encephalitis virus infection

**DOI:** 10.1186/s12974-019-1596-z

**Published:** 2019-11-07

**Authors:** Petra Pokorna Formanova, Martin Palus, Jiri Salat, Vaclav Hönig, Michal Stefanik, Pavel Svoboda, Daniel Ruzek

**Affiliations:** 1 Department of Virology, Veterinary Research Institute, Hudcova 70, CZ-62100 Brno, Czech Republic; 2 Institute of Parasitology, Biology Centre of the Czech Academy of Sciences, Branisovska 31, CZ-37005 Ceske Budejovice, Czech Republic; 3 Department of Chemistry and Biochemistry, Mendel University in Brno, Zemedelska 1, CZ-61300 Brno, Czech Republic

**Keywords:** Tick-borne encephalitis, Tick-borne encephalitis virus, Neuroinflammation, Luminex

## Abstract

**Background:**

Tick-borne encephalitis (TBE) is a severe neuropathological disorder caused by tick-borne encephalitis virus (TBEV). Brain TBEV infection is characterized by extensive pathological neuroinflammation. The mechanism by which TBEV causes CNS destruction remains unclear, but growing evidence suggests that it involves both direct neuronal damage by the virus infection and indirect damage caused by the immune response. Here, we aimed to examine the TBEV-infection-induced innate immune response in mice and in human neural cells. We also compared cytokine/chemokine communication between naïve and infected neuronal cells and astrocytes.

**Methods:**

We used a multiplexed Luminex system to measure multiple cytokines/chemokines and growth factors in mouse serum samples and brain tissue, and in human neuroblastoma cells (SK-N-SH) and primary cortical astrocytes (HBCA), which were infected with the highly pathogenic TBEV strain Hypr. We also investigated changes in cytokine/chemokine production in naïve HBCA cells treated with virus-free supernatants from TBEV-infected SK-N-SH cells and in naïve SK-N-SH cells treated with virus-free supernatants from TBEV-infected HBCA cells. Additionally, a plaque assay was performed to assess how cytokine/chemokine treatment influenced viral growth following TBEV infection.

**Results:**

TBEV-infected mice exhibited time-dependent increases in serum and brain tissue concentrations of multiple cytokines/chemokines (mainly CXCL10/IP-10, and also CXCL1, G-CSF, IL-6, and others). TBEV-infected SK-N-SH cells exhibited increased production of IL-8 and RANTES and downregulated MCP-1 and HGF. TBEV infection of HBCA cells activated production of a broad spectrum of pro-inflammatory cytokines, chemokines, and growth factors (mainly IL-6, IL-8, CXCL10, RANTES, and G-CSF) and downregulated the expression of VEGF. Treatment of SK-N-SH with supernatants from infected HBCA induced expression of a variety of chemokines and pro-inflammatory cytokines, reduced SK-N-SH mortality after TBEV infection, and decreased virus growth in these cells. Treatment of HBCA with supernatants from infected SK-N-SH had little effect on cytokine/chemokine/growth factor expression but reduced TBEV growth in these cells after infection.

**Conclusions:**

Our results indicated that both neurons and astrocytes are potential sources of pro-inflammatory cytokines in TBEV-infected brain tissue. Infected/activated astrocytes produce cytokines/chemokines that stimulate the innate neuronal immune response, limiting virus replication, and increasing survival of infected neurons.

## Introduction

Tick-borne encephalitis (TBE) is a severe and potentially lethal neurological infection caused by the TBE virus (TBEV), for which there are presently no specific and effective therapies available. Each year, over 13,000 TBE cases, and numerous TBE-related deaths, are reported in the large forested TBEV-endemic regions of Europe and Asia [1]. TBEV and several other medically important and emerging viral pathogens (e.g., yellow fever virus, West Nile virus, Japanese encephalitis virus, or Zika virus) are members of the genus *Flavivirus* within the family *Flaviviridae*, that constitute a group of small enveloped viruses with genomes comprising a single-stranded RNA of positive polarity [2]. TBEV is primarily transmitted to humans by *Ixodes* sp. ticks and also exhibits alimentary transmission following consumption of unpasteurized milk or milk products from TBEV-infected goats, sheep, and cows. The clinical presentation of TBE in humans ranges from mild flu-like fever to severe encephalitis or encephalomyelitis [1, 3]. The main target organ for TBEV is the host brain, where the virus primarily infects neurons [4]. However, other neural cells, such as astrocytes, are also reportedly susceptible to TBEV infection [5, 6]. Due to their anatomical association with the blood–brain barrier, astrocytes may constitute an important route through which TBEV penetrates the brain [7].

The outcome of TBE is largely determined by the balance between damage caused by viral replication and the immune response [8]. The mammalian immune system employs sophisticated antiviral measures to limit virus replication, but this process can have immunopathological consequences. The protective and pathological roles of the immune reaction must be balanced to support both efficient virus clearing and protection from immune system-mediated damage to brain tissue.

Immune response initiation and the recruitment of immune cells to the brain are primarily mediated by cytokines, chemokines, and interferons. Many studies describe the critical role of type I interferons (IFNs) in the defense against TBEV [9]. TBEV infection reportedly induces type I IFN expression in host cells, and the virus is highly sensitive to host cell pretreatment with IFNs [10–16]. In virus-infected cells and the surrounding tissues, the antiviral action of IFNs is mediated by the expression of hundreds of IFN-stimulated genes (ISG) [17]. Viperin is an important ISG that strongly inhibits TBEV, targeting the virus at multiple stages of its life cycle [9, 15, 16, 18–20]. However, little is known about the roles of soluble mediators other than IFNs, particularly other cytokines and chemokines, with regards to protection or immunopathology during TBE.

Our current knowledge about TBEV-associated cytokine and chemokine expression has been primarily obtained through analyses of human samples. Increased levels of various cytokines and chemokines have been detected in serum or cerebrospinal fluid (CSF) samples from TBE patients, and some of these cytokines/chemokines are considered biomarkers that indicate a severe course of infection. Human TBE patients exhibit elevated serum levels of the pro-inflammatory cytokines interleukin (IL)-6, IL-8, IL-12, IL-17A, and IL-17F and of the chemokines CCL3, CXCL2, CXCL10, and CXCL13 and elevated CSF levels of IL-8, IL-10, IL-17F, IL-22, CCL2, CCL3, CCL5/RANTES, CXCL1-2, and CXCL10-13 [21–27]. Compared to control patients, TBE patients exhibit higher ratios of IL-12:IL-4 and IL-12:IL-10, reflecting the global pro-inflammatory cytokine balance [28]. However, there remain unanswered questions regarding the dynamics of the production of these cytokines/chemokines during the course of TBE, about their antiviral or harmful roles and about the cell types that produce these immunologically important soluble factors within the host body.

In the present study, we aimed to assess the concentrations of different cytokines and chemokines in blood serum and brain tissue samples from mice at different time-points after infection with a lethal dose of TBEV. For these analyses, we employed a multiplex array using a fluorescent bead-based technology. We also used this system to investigate the TBEV-infection-evoked production of multiple cytokines and chemokines by human neural cells, and to compare the cytokine/chemokine communication and induction of antiviral response between naïve and infected neuronal cells and astrocytes.

## Methods

### Virus

The low-passage TBEV strain Hypr was provided by the Collection of Arboviruses, Institute of Parasitology, Biology Centre of the Czech Academy of Sciences, Ceske Budejovice, Czech Republic (http://www.arboviruscollection.cz/index.php?lang=en). Prior to its use in the in vitro experiments, the virus was propagated in BHK-21 cells and then purified by ultracentrifugation on a tartrate gradient as described previously [29]. In vivo experiments were performed using a mouse brain virus suspension. To generate ultraviolet (UV)-inactivated TBEV (mock), a viral suspension was incubated on ice for 15 min and exposed to UV light using a UV Crosslinker CL-508 (Uvitec Cambridge). Plaque assay was performed to verify inactivation of viral infectivity.

### Cells

Porcine kidney stable (PS) cells [30] were cultured at 37 °C in Leibovitz (L-15) medium with L-glutamine, supplemented with 3% fetal bovine serum, 100 U/mL penicillin, and 100 μg/mL streptomycin (Sigma-Aldrich, Czech Republic). Human neuroblastoma SK-N-SH cells (obtained from ATCC) were cultured at 37 °C in 5% CO_2_ in Dulbecco’s Modified Eagle’s Medium (DMEM) supplemented with 10% fetal bovine serum, 292 μg/mL L-glutamine, 100 U/mL penicillin, and 100 μg/mL streptomycin (Sigma-Aldrich, Czech Republic).

Human primary astrocytes (HBCA cells; purchased from ScienCell at passage 1) were grown following the supplier’s instructions in astrocyte medium with astrocyte growth supplement, 6% fetal bovine serum and 100 U/mL penicillin, and 100 μg/mL streptomycin (Sigma-Aldrich, Czech Republic). The cells exhibited typical astrocyte morphology and expressed GFAP, as confirmed by staining with GFAP antibody conjugated to Alexa Fluor 488 (1 : 100; Santa Cruz).

### Mice and virus inoculation

Female BALB/c mice of 5–6 weeks of age were obtained from Envigo, Inc. (Indiana, USA). The mice were housed in individually ventilated plastic cages (Techniplast) with sterilized wood-chip bedding, under a constant temperature of 22 °C and relative humidity of 65%. A diet of sterilized pellets and water was provided ad libitum.

The experiments included 25 mice per group. Mice in the experimental group were subcutaneously inoculated with approximately 500 plaque-forming units (pfu) of TBEV, while control mice received vehicle only. On days 1, 3, 5, 7, and 9 post-infection (p.i.), TBEV-infected (*n* = 5 mice/interval) and control (*n* = 5 mice/interval) mice were euthanized, and samples of serum, spleen, and brain were collected. Organs were individually weighted and then homogenized in sterile PBS using the TissueLyser II (Qiagen). The homogenate was clarified by centrifugation at 14,000×*g* for 10 min at 4 °C and then used for virus titration and/or measurement of cytokines and chemokines.

### Plaque assay

To determine the virus titer in cell culture supernatants, mouse sera, mouse spleen homogenate (17% w/v), and mouse brain homogenate (25% w/v), we performed plaque assays as previously described [31]. Briefly, to 24-well tissue culture plates, we added 10-fold dilutions of the virus plus a suspension of PS cells (1.3 × 10^5^ cells per well). After 4 h of incubation at 37 °C and 0.5% CO_2_, each well was overlaid with carboxymethylcellulose (1.5% in L-15 medium). After a 5-day incubation at 37 °C and 0.5% CO_2_, the cell monolayers were visualized using naphthalene black. Viral titers were expressed as plaque-forming units per milliliter.

### Measurement of cell viability

Cell viability was measured using Cell-Counting Kit-8 (CCK-8; Sigma-Aldrich) following the manufacturer’s instructions. Briefly, 10 μL CCK-8 solution was added to the cells in each well, followed by a 2-h incubation at 37 °C. Then, the absorbance at 450 nm was determined using an EL 808 Ultra microplate reader, and the data were processed with KC4 v. 3.1 software (BIO-TEK Instrumentations, Vermont, USA). Cell viability was expressed as the percentage of the reading for control (uninfected or untreated) cells.

### Infection of SK-N-SH and HBCA cells

In 96-well plates, SK-N-SH or HBCA cells were seeded at a concentration of 1 × 10^4^ cells/well to form a fully confluent monolayer for 1 day (SK-N-SH cells) or 3 days (HBCA cells). Then, these cells were infected with TBEV at a multiplicity of infection (m.o.i.) of 1. After 4 h of adsorption of the virus to the cells, the medium was removed, the cells were washed with virus-free medium, and fresh medium was added. Mock-infected cells were exposed to an equal dose of UV-inactivated TBEV. Cell-free culture supernatants were collected at the given time intervals and analyzed for virus titer using the above-described plaque assay or for analyzed for cytokine/chemokine concentrations as described below.

### Multiplex cytokine bead array assay

Cytokine and chemokine levels were measured in mouse serum (20× diluted) and brain samples (10× diluted) using the MILLIPLEX MAP Mouse Cytokine/Chemokine Magnetic Bead Panel (MCYTMAG-70 K-PX32; Millipore). Cytokine and chemokine levels were measured in human cell culture supernatants using the Human Cytokine Magnetic 30-Plex Panel for the Luminex platform (Life Technologies, Frederick, MD). All measurements were performed using a MAGPIX instrument (Luminex, Austin, TX), following the manufacturer’s instructions. Data were collected using xPonent software (Luminex), log transformed, and analyzed as described below.

### Treatment of naïve SK-N-SH cells with supernatants from TBEV-infected HBCA

Supernatants from TBEV-infected and mock-infected HBCA cells were collected at 1 and 5 days p.i. The harvested supernatants were cleared of the virus using a Vivaspin centricone with a 100-kD membrane (Sartorious), and successful virus removal was confirmed by plaque assay. SK-N-SH cells were cultivated in 96-well plates, and then incubated with 100 μL of the HBCA supernatants for 4 h. Next, the cells were washed twice with PBS and fresh medium was added. On days 1 and 3 post-treatment, we measured the cell viability and collected the cell culture supernatants for cytokine/chemokine measurements as described above.

### Treatment of naïve HBCA cells with supernatants from TBEV-infected SK-N-SH

Supernatants from TBEV-infected and mock-infected SK-N-SH cells were collected at 1 and 3 days p.i. The harvested supernatants were cleared of virus using the Vivaspin centricone with a 100-kD membrane (Sartorious), and successful virus removal was confirmed by plaque assay. HBCA cells were cultured in 96-well plates and incubated with 100 μL of the SK-N-SH supernatants for 4 h. Then, the cells were washed twice with PBS and fresh medium was added. On days 1 and 5 post-treatment, we measured the cell viability, and collected the cell culture supernatants for cytokine/chemokine measurements as described above.

### Pretreatment of TBEV-infected SK-N-SH cells with supernatants from TBEV-infected HBCA

Supernatants from TBEV-infected and mock-infected HBCA cells were collected at 1 and 5 days p.i. and were cleared of virus using the Vivaspin centricone with a 100-kD membrane (Sartorius). Successful virus removal was confirmed by plaque assay. SK-N-SH cells were cultivated in 96-well plates and incubated with 100 μL of the HBCA supernatants for 4 h. Then, the cells were washed twice with PBS and fresh medium containing TBEV (m.o.i. = 1) was added. On days 1 and 3 post-treatment, the cell viability was measured as described above and the cell culture supernatants were collected for viral titer measurement by plaque assay.

### Pretreatment of TBEV-infected HBCA cells with supernatants from TBEV-infected SK-N-SH

Supernatants from TBEV-infected and mock-infected SK-N-SH cells were collected at 1 and 3 days p.i. The harvested supernatants were cleared of virus using the Vivaspin centricone with a 100-kD membrane (Sartorius), and successful virus removal was confirmed by plaque assay. HBCA cells were cultured in 96-well plates, and incubated with 100 μl of the SK-N-SH supernatants for 4 h. Then, the cells were washed twice with PBS, and fresh medium containing TBEV (m.o.i. = 1) was added. On days 1 and 5 post-treatment, the cell viability was measured as described above and the cell culture supernatants were collected for viral titer measurement by plaque assay as described above.

### Data processing and statistical analyses

The data in graphs are expressed as the mean ± standard error. The significance of between-group differences was evaluated by unpaired Student’s *t* test. The data from the multiplex cytokine bead array assay from the in vitro experiments were log transformed and analyzed by multiple *t* test, with false discovery rate correction comprising the two-stage linear step-up procedure of Benjamini, Krieger, and Yekutieli (*Q*, 1%). From the mouse experiment, the data from the multiplex cytokine bead array assay were log transformed and analyzed by multiple *t* tests, with correction for multiple comparisons using the Holm-Sidak method, with alpha = 0.05. All analyses were performed using GraphPad Prism 7, version 7.04 (GraphPad Software, Inc.). Differences with *p* < 0.05 were considered significant.

## Results

### Virus load in serum, the spleen, and the brain of TBEV-infected mice

**Fig. 1:**
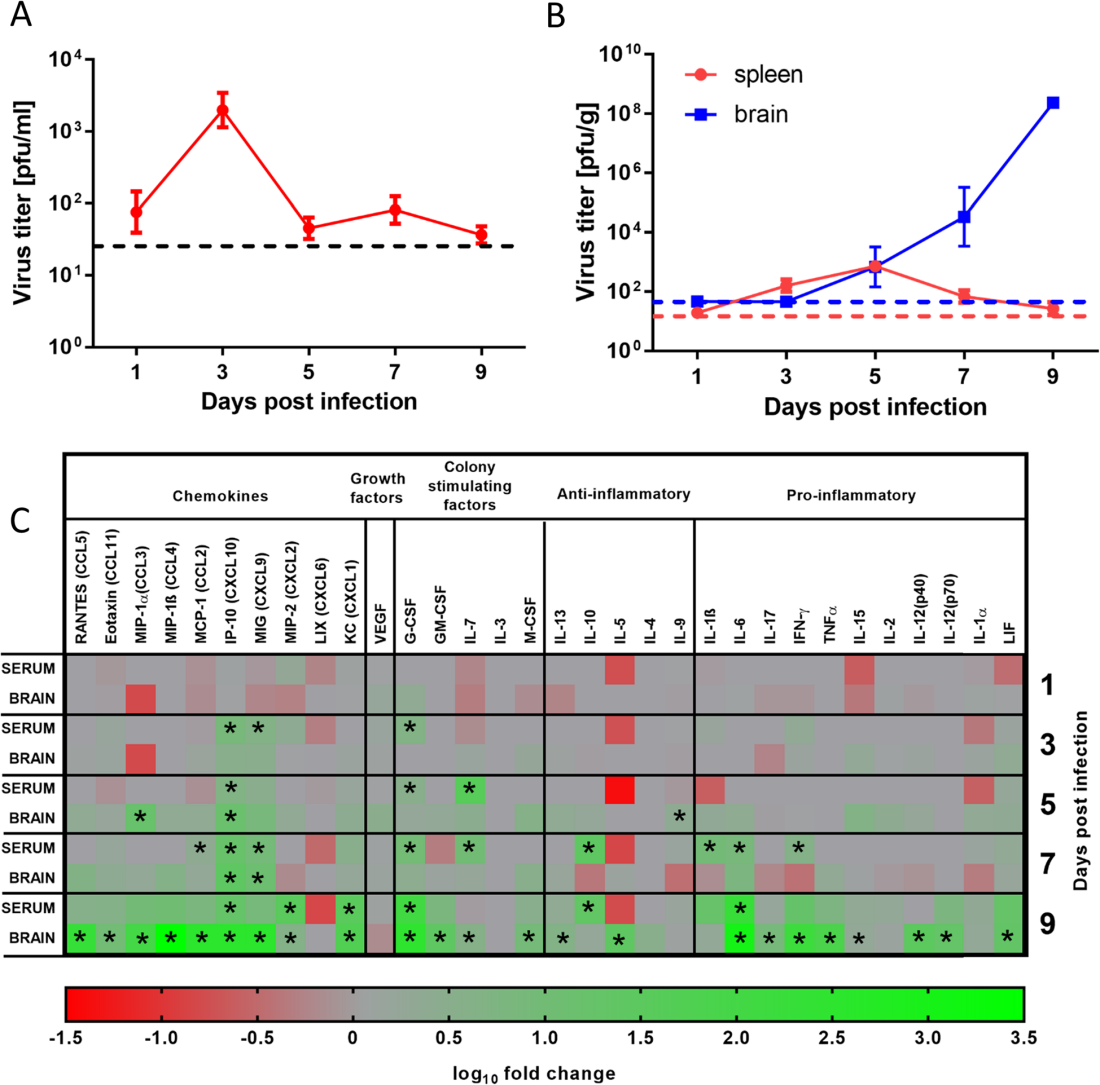
Virus titer and cytokine/chemokine levels in the serum and organs of tick-borne encephalitis virus (TBEV)-infected mice. Female BALB/c mice were subcutaneously inoculated with ~ 500 pfu of TBEV and then euthanized on days 1, 3, 5, 7, and 9 p.i. Serum, spleen, and brain samples were collected and used for virus titration and cytokine/chemokine measurement. **a** Virus titer in serum samples from infected mice (dashed line; detection threshold, 1.2 log_10_ pfu/mL). **b** Virus titer in spleen and brain tissue suspensions (dashed line; detection threshold for brain, 1.8 log_10_ pfu/g and for spleen, 1.2 log_10_ pfu/g). **c** The levels of multiple cytokines, chemokines, colony-stimulating factors, and growth factors were determined using the MILLIPLEX MAP Mouse Cytokine/Chemokine Magnetic Bead Panel assay. Asterisks indicate statistically significant differences compared to uninfected controls

Serum samples were harvested from TBEV-infected BALB/c mice at 1, 3, 5, 7, and 9 days p.i. The virus titers in individual samples were determined by plaque assay on PS cells. Peak viremia was observed on day 3 p.i., reaching approximately 3.2 log_10_ pfu/mL. On days 1, 5, 7 and 9 p.i., the serum TBEV titer was low (< 2 log_10_ pfu/mL)—only slightly above the plaque assay’s detection limit (Fig. 1a).

Spleen and brain samples were collected from the TBEV-infected BALB/c mice at 1, 3, 5, 7, and 9 days p.i. Spleen samples showed low TBEV titers at all investigated time-points, with the peak titer observed on day 5 p.i., reaching approximately 2.5 log_10_ pfu/g. Brain samples collected on days 1 and 3 p.i. exhibited no virus. TBEV was first detected in brain tissue on day 5 p.i., and the titers increased over the subsequent time-points. The peak virus titer in brain tissue was observed on day 9, reaching 8.3 log_10_ pfu/g. This time-point was also associated with the first clinical signs of neuroinfection, including ruffled fur, apathy, tremor, and hunched posture (Fig. 1b).

### Analysis of cytokine/chemokine levels in serum of TBEV-infected mice

**Fig. 2:**
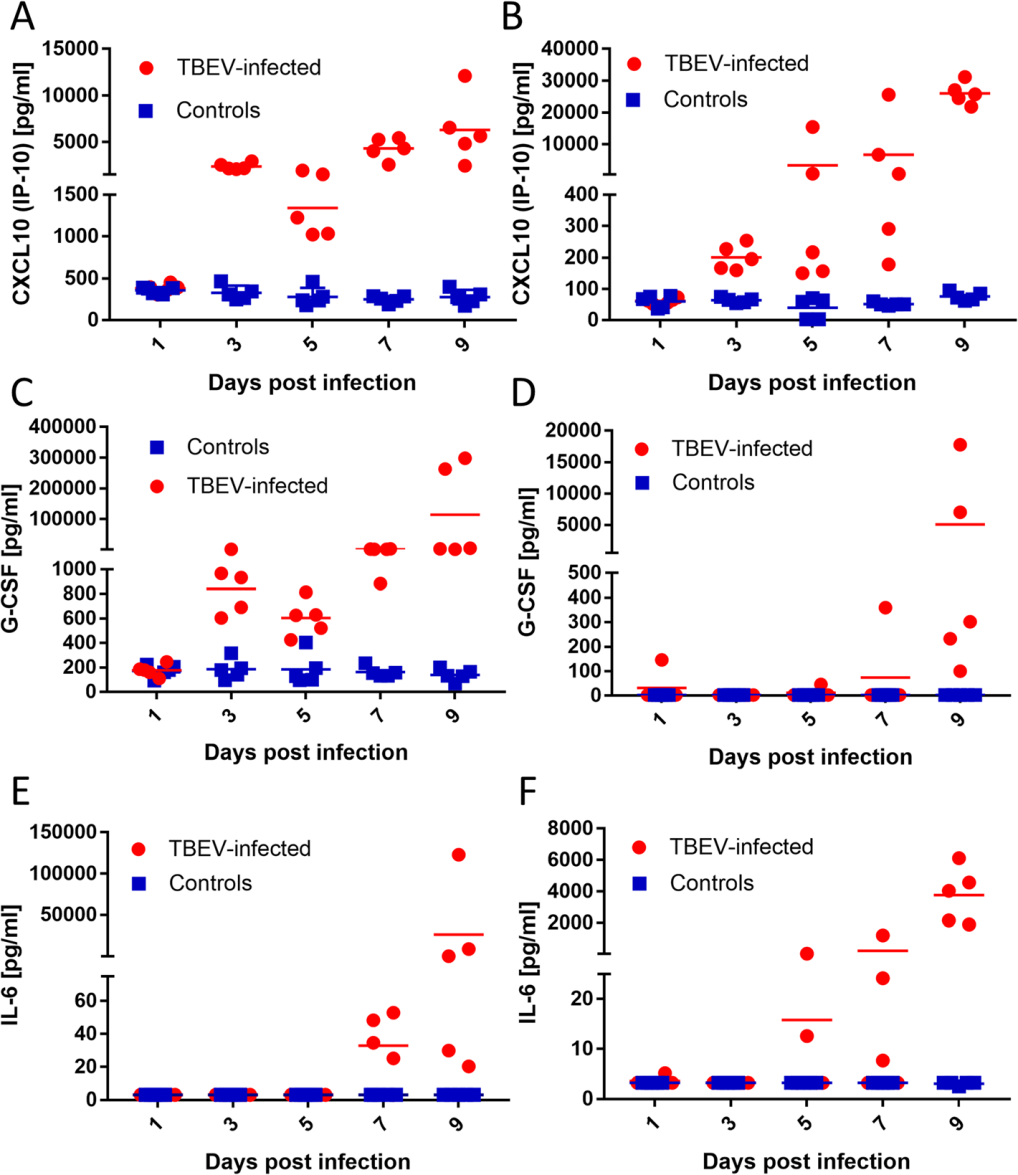
Dynamics of the production of selected cytokines/chemokines in the serum (**a**, **c**, **e**) and brain tissue (**b**, **d**, **f**) of mice infected with tick-borne encephalitis virus (TBEV). Female BALB/c mice were subcutaneously inoculated with ~ 500 pfu of TBEV, and then euthanized on days 1, 3, 5, 7, and 9 p.i. Serum and brain samples were collected and used for measurement of cytokine/chemokine levels with the MILLIPLEX MAP Mouse Cytokine/Chemokine Magnetic Bead Panel assay. The dynamics of production of CXCL10 (**a**, **b**), G-CSF (**c**, **d**), and IL-6 (**e**, **f**) are shown

Using the multiplex cytokine bead array assay, we analyzed 32 cytokines/chemokines in serum samples from TBEV-infected and mock-infected mice at days 1, 3, 5, 7, and 9 p.i. On day 1 p.i., no cytokine or chemokine concentrations differed between TBEV-infected and mock-infected mice. On day 3 p.i. (i.e., at the time of peak of viremia), TBEV-infected mice showed significantly increased concentrations of the chemokines CXCL10/IP-10 and CXCL9/MIG, and the colony-stimulating factor G-CSF. TBEV-infected mice showed continuously increased or gradually increasing concentrations of CXCL10 and G-CSF throughout the remaining duration of the experiment (Figs. 1c and 2a). Thus, CXCL10 and G-CSF were the earliest and most robustly upregulated chemokines in the serum of TBEV-infected animals, with elevation detectable starting at the viremic phase of infection.

On day 5 p.i., TBEV-infected mice exhibited increased serum concentrations of CXCL10, and the colony-stimulating factors IL-7 and G-CSF. On day 7 p.i., TBEV-infected mice had increased serum levels of the chemokines CXCL10, CCL2/MCP-1, and CXCL9; the colony-stimulating factors G-CSF and IL-7; and the pro-inflammatory cytokines IL-10, IFN-γ, IL-1β, and IL-6. On day 9 p.i. (i.e., the start of the neurological phase of the disease) and TBEV-infected mice exhibited increased levels of CXCL10, CXCL1/KC, CXCL2, G-CSF, IL-10, and IL-6 (Figs. 1c and 2a, c and e).

### Analysis of cytokine/chemokine levels in brains of TBEV-infected mice

Brain tissues were collected from TBEV-infected and mock-infected mice on days 1, 3, 5, 7, and 9 p.i. These samples were homogenized and analyzed for the concentrations of 32 cytokines/chemokines using the multiplex cytokine bead array assay. On days 1 and 3 (i.e., before the virus reached the brain), the cytokine and chemokine levels in the brain did not differ between TBEV- and mock-infected mice. On day 5 p.i. (i.e., at the time of neuroinvasion, when only low virus titers were detected in the brain), TBEV-infected mice exhibited increased levels of CXCL10 and MIP-1α in brain tissue. This increased CXCL10 production was robust and stable for the rest of the experiment (Fig. 2b). On day 7 p.i., CXCL10 continued to be the main chemokine increased in the brain tissue of TBEV-infected mice. Infected brains also showed a slight but statistically significant increase of CXCL9 at this time-point. On day 9 p.i. (i.e., when mice had high TBEV titers in the brain and showed neurological signs of infection), the TBEV-infected mice showed increased levels of numerous chemokines, colony-stimulating factors, and pro-inflammatory cytokines compared to mock-infected brain tissues (Fig. 1c). TBEV-infected brains also exhibited slight but statistically significant increases of the anti-inflammatory cytokines IL-5 and IL-13 (Fig. 1c).

### Growth of TBEV in SK-N-SH and HBCA cells

**Fig. 3:**
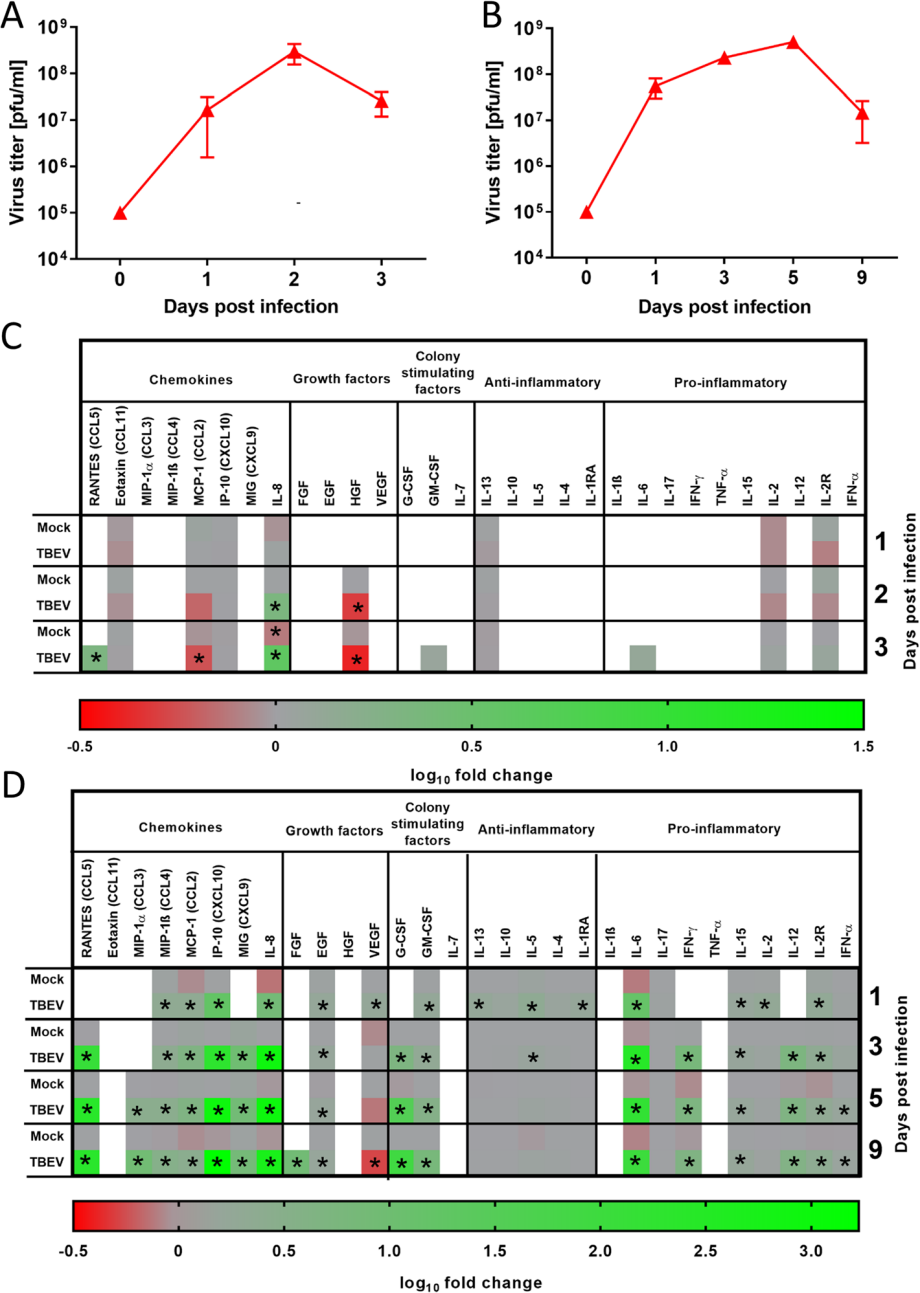
Growth of tick-borne encephalitis virus (TBEV) and cytokine/chemokine production in SK-N-SH and HBCA cells. SK-N-SH or HBCA cells were infected with TBEV (m.o.i. = 1), and then cell-free culture supernatants were collected at given time intervals. Supernatants were analyzed for virus titer using a plaque assay or analyzed for cytokine/chemokine concentration. TBEV growth in SK-N-SH cells (**a**) and HBCA cells (**b**). Supernatants from TBEV-infected, uninfected, and mock-infected (UV-inactivated virus) SK-N-SH cells (**c**) and HBCA cells (**d**) were analyzed for levels of multiple cytokines, chemokines, and growth factors using the Human Cytokine Magnetic 30-Plex Panel for the Luminex platform. Asterisks indicate statistically significant differences compared to uninfected controls

SK-N-SH cells were infected with TBEV (m.o.i. = 1) and cell culture supernatants were collected at 0, 1, 2, and 3 days p.i. The virus titer was assayed by plaque assay, revealing that the highest increase in virus production occurred between days 0 and 2 p.i., reaching a peak titer of approximately 8.5 log_10_ pfu/mL. A gross cytopathogenic effect (CPE) was observed on day 3 p.i., corresponding with a virus titer decrease to approximately 7.4 log_10_ pfu/mL (Fig. 3a).

HBCA cells were infected with TBEV (m.o.i. = 1), and cell culture supernatants were collected at 0, 1, 3, 5, and 9 days p.i. Virus titer was assayed by plaque assay, revealing that the highest increase in virus production occurred between 0 and 1 day p.i., reaching a virus titer of approximately 7.7 log_10_ pfu/mL. The virus titer additionally increased up to day 5 p.i., reaching a peak value of 8.7 log_10_ pfu/mL. Between days 5 and 9 p.i., the virus titer decreased by about 1.5 log_10_ pfu/mL (Fig. 3b). Infected HBCA showed little CPE with isolated rounded cells.

### Cytokines/chemokines produced by TBEV-infected SK-N-SH cells

SK-N-SH cells were infected with TBEV (m.o.i. = 1), and mock-infected cells were treated with the same dose of UV-inactivated TBEV. Cell culture supernatants were collected at 1, 2, and 3 days p.i. from TBEV-infected, mock-infected, and uninfected cells. We measured the concentrations of 30 cytokines and chemokines in the collected supernatants using the multiplex cytokine bead array assay. Most of the analytes (19 of 30) were at undetectable levels at all investigated time-points (Fig. 3c, white boxes). TBEV-infected cells showed increased production of IL-8 at 2 days p.i. and of IL-8 and RANTES at 3 days p.i. TBEV-infected cells also exhibited downregulated expression of HGF at 2 and 3 days p.i., and of MCP-1 at day 3 p.i. (Fig. 3c).

### Cytokines/chemokines produced by TBEV-infected HBCA cells

TBEV infection of HBCA cells (m.o.i. = 1) elicited production of a broad array of chemokines, growth factors, colony-stimulating factors, and mainly pro-inflammatory cytokines, as measured by multiplex cytokine bead array assay. Cytokine/chemokine production did not significantly differ in HBCA cells treated with UV-inactivated TBEV compared to uninfected HBCA cells (Fig. 3d). As early as 1 day p.i., TBEV infection activated production of several chemokines (e.g., CXCL10 and IL-8), growth factors (EGF and VEGF), and pro-inflammatory cytokines (predominantly IL-6). At the subsequent time-points, we observed further increased concentrations of these analytes in the culture supernatants of TBEV-infected HBCA cells. Over all investigated time-points, TBEV-infected HBCA cells showed dominant production of RANTES, CXCL10, IL8, G-CSF, and IL-6 (Fig. 3d).

### Supernatants from TBEV-infected SK-N-SH have little effect on cytokine/chemokine production by HBCA cells but reduce viral growth and increase cell survival of TBEV-infected astrocytes

**Fig. 4:**
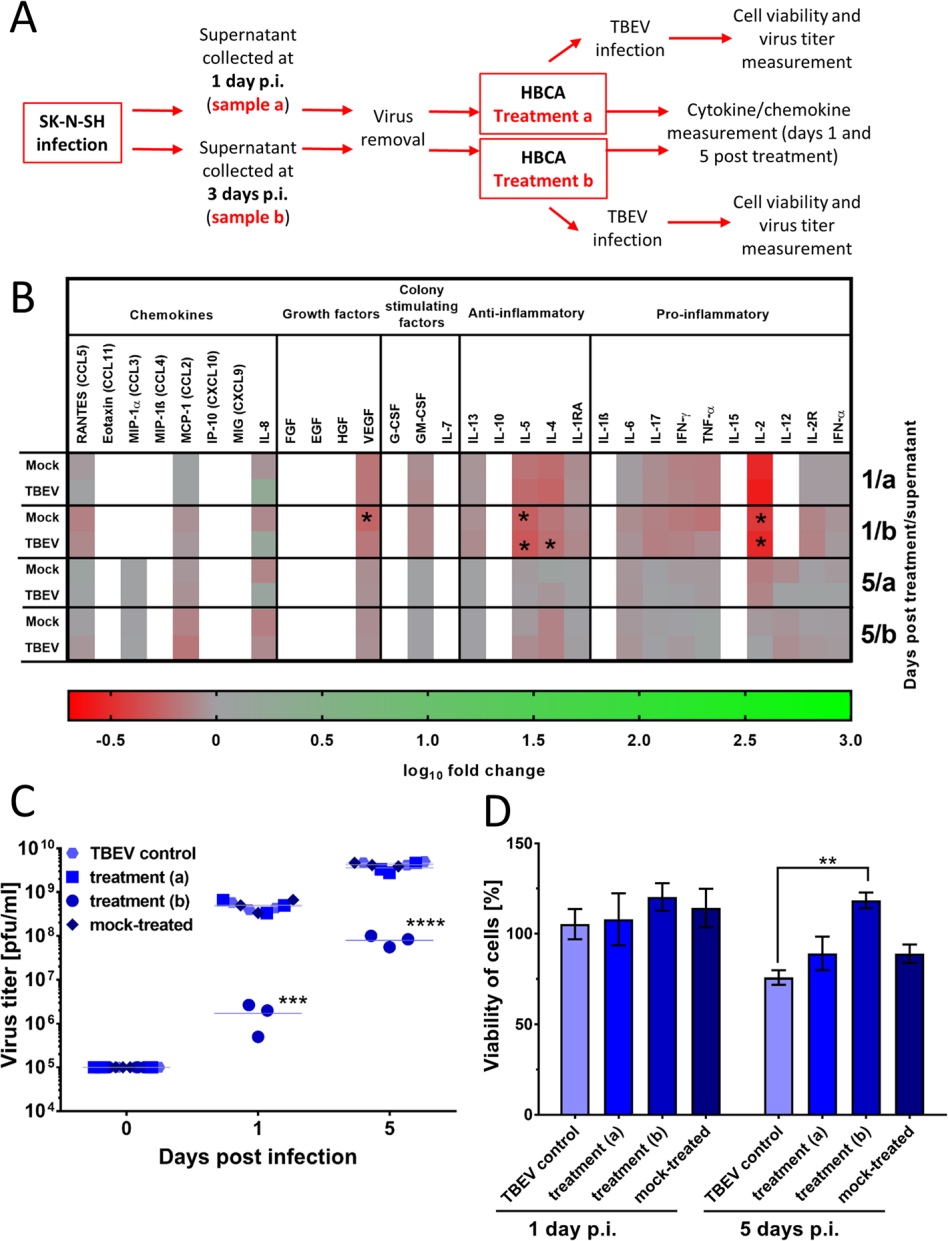
Treatment of naïve HBCA cells with supernatants from tick-borne encephalitis virus (TBEV)-infected SK-N-SH. **a** Schema of the experiment. Supernatants from TBEV-infected and mock-infected SK-N-SH cells were collected at days 1 (treatment a) and 3 (treatment b) p.i. and cleared of the virus. Then, HBCA were incubated with the virus-free SK-N-SH supernatants for 4 h. On days 1 and 5 post-treatment, the cell culture supernatants were collected and used for cytokine/chemokine measurements with the Human Cytokine Magnetic 30-Plex Panel for the Luminex platform. Asterisks indicate statistically significant differences compared to uninfected controls (**b**). HBCA cells were incubated with SK-N-SH supernatants for 4 h and then infected with TBEV (m.o.i. = 1). On days 0, 1, and 5 p.i., supernatants were collected and used for determination of viral titer (**c**). Cell viability was also assessed (**d**). **p < 0.01; ***p < 0.001; ****p < 0.0001

To investigate how soluble factors produced by TBEV-infected SK-N-SH cells affected cytokine/chemokine production by astrocytes, we collected culture media from TBEV-infected, mock-infected, and uninfected SK-N-SH cells on days 1 and 3 p.i. The collected media were cleared of virus and used to treat naïve astrocytes for 4 h. After 1 and 5 days post-treatment, cell culture media were collected and expression of cytokines/chemokines was analyzed (Fig. 4a). As shown in Fig. 4b, treatment of naïve astrocytes with culture media from TBEV-infected neuroblastoma cells collected on day 1 p.i. did not lead to increased production of any of the analyzed soluble factors. When treated with media from either TBEV- or mock-infected SK-N-SH cells collected on day 3 p.i., astrocytes exhibited significantly decreased production of IL-2 and IL-5 at 1 day post-treatment. These astrocytes also exhibited significantly decreased IL-4 production, but only after treatment with media from TBEV-infected SK-N-SH cells (Fig. 4b).

Next, we investigated how soluble factors produced by TBEV-infected SK-N-SH cells influenced cell survival and viral growth in TBEV-infected astrocytes. HBCA cells were treated for 4 h with cell culture media collected from TBEV-infected SK-N-SH cells. Then, the treated HBCA cells were infected with TBEV (m.o.i. = 1), and viral growth and cell viability were measured. Treatment of HBCA cells with SK-N-SH media collected at day 1 p.i. did not influence viral growth or cell viability at any investigated time-points (Fig. 4c and d). In contrast, treatment of HBCA cells with SK-N-SH media collected at day 3 p.i. significantly reduced TBEV growth in the infected HBCA cells at both days 1 and 5 p.i. (Fig. 4c). Compared to untreated cultures, treatment increased the viability of TBEV-infected HBCA cells at day 5 p.i. (Fig. 4d; *p* < 0.01).

### Supernatants from TBEV-infected HBCA activate cytokine/chemokine production by SK-N-SH cells, reduce viral growth, and increase survival of TBEV-infected SK-N-SH

**Fig. 5:**
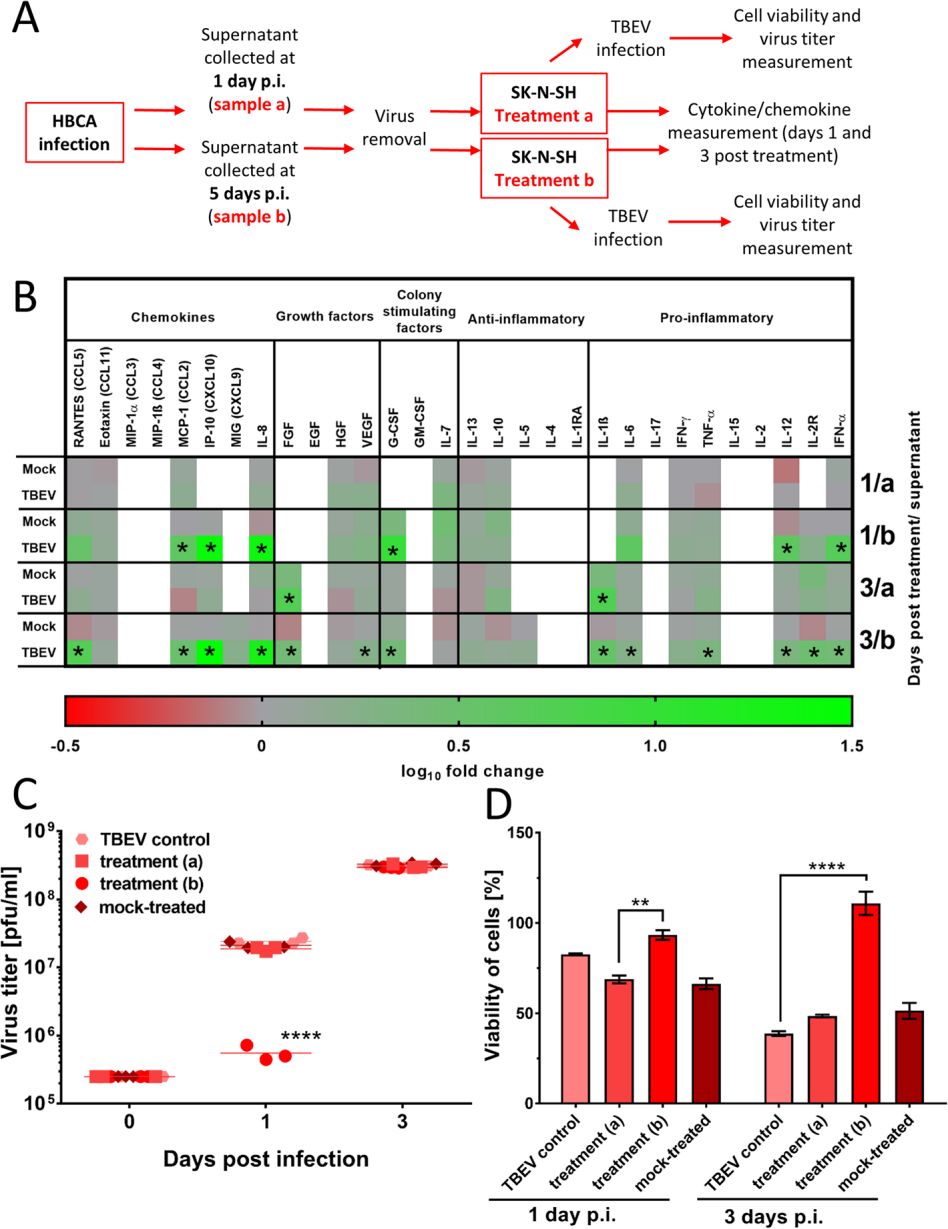
Treatment of naïve SK-N-SH cells with supernatants from tick-borne encephalitis virus (TBEV)-infected HBCA. **a** Schema of the experiment. Supernatants from TBEV-infected and mock-infected HBCA cells were collected at days 1 (treatment a) and 5 (treatment b) p.i. and then cleared of the virus. SK-N-SH were incubated with the virus-free HBCA supernatants for 4 h. On days 1 and 3 post-treatment, the cell culture supernatants were collected and used for cytokine/chemokine measurements with the Human Cytokine Magnetic 30-Plex Panel for the Luminex platform. Asterisks indicate statistically significant differences compared to uninfected controls (**b**). SK-N-SH cells were incubated with the HBCA supernatants for 4 h and then infected with TBEV (m.o.i. = 1). On days 0, 1, and 3 p.i., the supernatants were collected and used for determination of viral titer (**c**). The cell viability was also assessed (**d**). **p < 0.01; ***p < 0.001; ****p < 0.0001

We next investigated how the chemokines and cytokines released from TBEV-infected HBCA cells contributed to mediating inflammatory cytokine and chemokine responses in SK-N-SH cells. Supernatant media from TBEV-infected and mock-infected HBCA cultures were collected at days 1 and 5 p.i., cleared of the virus and used to treat naïve SK-N-SH cells for 4 h (Fig. 5a). As depicted in Fig. 5b, cytokine and chemokine production levels of SK-N-SH cells at all investigated time-points were not affected by treatment with media collected from mock-infected HBCA, or treatment with culture supernatants collected from TBEV-infected HBCA cells at 1 day p.i. In contrast, treatment of SK-N-SH with supernatants collected from TBEV-infected HBCA at day 5 resulted in significantly increased production of several cytokines and chemokines—particularly the chemokines CXCL10 and IL-8, the growth factors FGF and G-CSF, and a number of pro-inflammatory cytokines (Fig. 5b).

Finally, naïve SK-N-SH cells were treated for 4 h with supernatant media collected from TBEV- and mock-infected HBCA cultures and then infected with TBEV (m.o.i. = 1). Treatment of SK-N-SH with supernatants from mock-infected HBCA (collected at 1 and 5 days p.i.) or from TBEV-infected HBCA (collected at 1 day p.i.) did not affect viral growth or cell viability. In contrast, following treatment with media from TBEV-infected HBCA collected at 5 days p.i., SK-N-SH cells exhibited significantly reduced viral growth and increased cell viability compared to untreated controls (Fig. 5c and d).

## Discussion

TBEV infection primarily targets neurons, leading to their damage and death [3]. Accumulating evidence suggests that some neuronal damage may also be mediated by the immune response itself [32]. CNS infection leads to T cell recruitment, followed by cytotoxic T cell-induced immunopathogenesis [32]. Chemokines help regulate leukocyte trafficking into the CNS. Leukocytes are not only essential components of cell-mediated immunity and antiviral host defense, but also of immune system-mediated pathology [21]. To date, little is known about the dynamics of cytokine/chemokine production in serum and brain tissue or about the relative contributions of resident CNS cells (e.g., neurons and astrocytes) to inflammation development during TBE. The results of our present study yielded four major findings. First, CXCL10 and G-CSF are the earliest and most robustly upregulated chemokines, exhibiting increased presence in the serum of TBEV-infected mice as early as during the viremic phase. Second, the TBEV-infected brain shows increased levels of numerous chemokines, colony-stimulating factors, and pro-inflammatory cytokines, and these increases correlate with virus growth in the infected brain tissue. Third, TBEV-infected astrocytes, and to a lesser extent neuronal cells, are a potential source of pro-inflammatory cytokines/chemokines in the CNS. Fourth, cytokines/chemokines produced by TBEV-infected astrocytes or neuronal cells reduce virus growth in neuronal cells and increase the survival of infected neuronal cells or astrocytes, respectively.

In the mouse model of TBE, which recapitulates pathogenesis observed in severe/lethal cases of TBE in humans [33], we measured the cytokine/chemokine levels in serum and brain tissue at different stages of infection. In agreement with previous studies [34, 35], peak viremia occurred on day 3 p.i., and the viremic phase was followed by the virus reaching the brain, where the virus titer continuously increased until the end of the experiment. The titers reached in mouse sera are comparable with titers seen in our previous studies [32–35], and TBEV RNA levels seen in sera of human patients collected in the first phase of the disease [36, 37]. CXCL10 and G-CSF were the earliest and most robustly upregulated chemokines, showing an increased presence in the serum of TBEV-infected mice as early as during the viremic phase. A prior evaluation of chemokine concentrations in the serum of patients with TBE and a control group indicated that CXCL10 may be a good serum biomarker of TBEV-induced CNS inflammation [21]. Our present study demonstrated that increased serum levels of CXCL10 appeared before the virus reached the brain, possibly reflecting viral infection of peripheral tissues and organs during the first phase of disease; however, the exact source of this chemokine remains elusive. On days 7 and 9 p.i., we observed increased concentrations of IL-6 and IL-10. This is in agreement with prior analyses of serum samples from TBE patients, which have revealed increased serum levels of IL-6 [28, 38, 39] and IL-10 at admission, with these increases remaining for 4 weeks after treatment [38, 39]. Overall, comparisons of our recent results in a mouse model with the findings from previous studies of human TBE patients reveal that CXCL10 is the main serum biomarker associated with TBE in both TBEV-infected mice and humans.

Increased CXCL10 levels were detectable in the brain tissue of TBEV-infected mice at the time of viremia and viral neuroinvasion. During these stages of infection, little or no virus could be detected in the brain; thus, the elevated CXCL10 in the brain at this time was likely of peripheral origin. At later time-points, as the neuroinfection progressed, CXCL10 levels in the brain continued to substantially increase. Since serum CXCL10 levels remained relatively stable across these time-points, the increased CXCL10 level in the brain was most likely of neural origin. Increased CXCL10 concentrations have also been reported in the CSF of human TBE patients [21, 22, 27], where CXCL10 plays roles in recruiting CXCR3-expressing T cells into the brain/CSF [27, 40]. The recruitment of antigen-specific CD8^+^ T lymphocytes into the brain parenchyma is a key step in clearing viral infection from the brain [41, 42]; however, these cells can also play pathological roles under some circumstances [32]. Previous studies by our group [34] and others [43–46] using different viruses and models have demonstrated that excessively high CXCL10 levels in the CNS can be harmful to the host. For example, a study of mice with different susceptibilities to TBEV infection revealed that higher CXCL10 expression in the brain correlated with more severe course of infection and poorer outcome [34]. CXCL10 can cause pathology through excessive recruitment of cytotoxic T cells, and by cytotoxic action on neurons [47].

On day 9 p.i., which was the time of advanced brain infection, infected mouse brains exhibited increased levels of numerous other chemokines, colony-stimulating factors, and pro-inflammatory cytokines. This is in full agreement with the findings of previous studies in mice [34, 35, 48] and of studies analyzing CSF samples from patients with TBE [21, 23, 49, 50]. CSF samples from children or adult TBE patients have exhibited higher levels of CXCL1 [25], IFN-γ, IL-4, IL-6, IL-8 [51], CXCL10, CXCL11, CXCL12, CXCL13 [21], CCL2 [50], CCL3 [49], and other pro-inflammatory soluble factors. Additionally, increased concentrations of CCL5 (RANTES) and IL-5 have been detected in the CSF, but not in the serum, of patients with TBE [23, 26]. Similarly, in the present study, TBEV-infected mice showed increased concentrations of CCL5 (RANTES) and IL-5 on day 9 p.i. only in the brain tissue, with no changes in the serum levels of these molecules throughout the experiment. This is also in agreement with the findings of Zhang et al. [52]. Antagonizing RANTES within the CNS reportedly extended the survival of TBEV-infected mice and reduced the accumulation of infiltrating cells in the brain after TBEV infection [52], indicating that CCL5 (RANTES) causes neuroinflammation and may contribute to brain destruction during TBE [52, 53]. In summary, TBEV infection elicits strong production of various cytokines, chemokines, and colony-stimulating factors in mouse brains. For most of these soluble factors, the results in TBEV-infected mice show a good correlation with the findings in human CSF samples. CXCL10 is one of the earliest and most robustly upregulated chemokines in the TBEV-infected brain.

We next investigated the relative contributions of major resident CNS cells—specifically neuronal cells and astrocytes (HBCA)—as mediators of brain inflammation, by examining the ability of TBEV-infected human neuroblastoma cells (SK-N-SH) to produce key cytokines and chemokines. In response to TBEV infection, SK-N-SH exhibited increased production of IL-8 and CCL5 (RANTES). In contrast, infection of SK-N-SH with WNV reportedly results in a sharp increase of key pro-inflammatory cytokines (e.g., IL-1β, IL-6, IL-8, and TNF-α) at day 2 p.i., which coincides with peak virus replication [54]. It seems likely that neurons may be a main source of cytokines in WNV-associated neuroinflammation [54]. In contrast, TBEV-infected neuronal cells seem to contribute less to the development of brain inflammation in TBE compared to WNV encephalitis.

In our previous study, we found that HBCA were permissive to TBEV infection and that infection with the low-pathogenic TBEV strain Neudoerfl induced markedly increased expression of glial fibrillary acidic protein (GFAP), which is a marker of astrocyte activation [5]. We also observed the upregulated production of matrix metalloproteinase 9, and the upregulated mRNA expression and/or protein production of several key pro-inflammatory cytokines/chemokines, including TNF-α, IFNα, IL-1β, IL-6, IL-8, CXCL10, and CCL4, but not MCP-1 [5]. In our present study, we investigated the production of a broader panel of cytokines/chemokines in HBCA infected with the highly pathogenic TBEV strain Hypr. While HBCA infection with Neudoerfl and primary rat astrocyte infection with the Ljubljana I strain do not affects cell viability [5, 6], infection of HBCA with Hypr led to a slightly advanced CPE and cell death. Our results showed that Hypr infection of HBCA induced increased production of numerous cytokines/chemokines—particularly CCL5 (RANTES), CXCL10, IL-6, and IL-8, which confirmed our previous findings. While TNF-α mRNA expression was upregulated in HBCA infected with the Neudoerfl strain [5], we found that TNF-α remained at undetectable levels in HBCA cultures infected with Hypr strains. Additionally, MCP-1 mRNA expression was not upregulated in Neudoerfl-infected HBCA [5], but MCP-1 protein expression was upregulated in Hypr-infected HBCA.

Other studies have also reported that TBEV infection upregulates CCL5 (RANTES) production at both the mRNA and protein levels in primary astrocytes [52, 53]. Specifically, RANTES production in human primary astrocytes is induced by viral NS5 (particularly the RNA-dependent RNA polymerase domain), but not by other proteins of TBEV. It appears that TBEV NS5 activates the IFN regulatory factor 3 (IRF-3) signaling pathway in a manner dependent on RIG-I/MDA5, which causes the nuclear translocation of IRF-3, and its subsequent binding with the RANTES promoter [53]. As mentioned above, the CCL5 (RANTES)-mediated migration of blood monocytes and T lymphocytes may contribute to brain damage during TBE [53]. HBCA infection with another flavivirus, Zika virus, is associated with only limited activation of immune cytokine/chemokine response, but ZIKV infection induces the highest upregulation of CXCL10, IL-6, 8, 12, and CCL5 (RANTES) [55], which corresponds with the observations in TBEV-infected HBCA. Overall, the available data indicate that astrocytes are an important source of various pro-inflammatory cytokines and chemokines in the TBEV-infected brain. Some of these soluble factors, such as CXCL10 and CCL5 (RANTES), are known to mediate neuronal damage, which can have important pathological consequences during TBE.

We next investigated how the pro-inflammatory cytokines released from TBEV-infected SK-N-SH cells influenced naïve HBCA. Surprisingly, treatment of naïve HBCA with virus-free culture media from TBEV-infected neuroblastoma cells did not increase the production of any of the analyzed soluble factors. A study of WNV demonstrated that neurotoxic mediators released from infected SK-N-SH can activate astrocytes, as measured by a significant increase in GFAP expression, which was also associated with significant upregulation of inflammatory cytokines in naïve astrocytes [54]. On the other hand, we found that treatment of naïve SK-N-SH with virus-free culture media from TBEV-infected astrocytes resulted in increased production of several cytokines and chemokines—particularly the chemokines CXCL10 and IL-8, the growth factors FGF and G-CSF, and a number of pro-inflammatory cytokines. While TBEV infection of SK-N-SH increased the expression of only two soluble factors (IL-8 and CCL5), treatment of the same cells with cytokines/chemokines produced by TBEV-infected HBCA induced much stronger immune activation. Although TBEV-infected HBCA produced some soluble factors with known neurotoxic properties [47, 51, 54], supernatants derived from these cells caused no toxicity towards treated SK-N-SH cells. Notably, this result does not necessarily mean that cytokines/chemokines exert no neurotoxicity during TBE; it is most likely related to the concentration used in our experiments. This issue warrants further experimentation, ideally with primary neurons, which is beyond the aim and purpose of our present study. Interestingly, treatment of SK-N-SH with supernatants from TBEV-infected HBCA (at early time-points), and treatment of HBCA with supernatants from TBEV-infected SK-N-SH led to reduced viral growth and increased survival of the infected cells. Our data do not enable us to pinpoint the specific mediator derived from infected SK-N-SH or HBCA that contributed to reducing viral growth and virus-induced cell mortality; however, CXCL10, RANTES, IL-8, and IL-6 are likely candidates. Our next investigations will focus on the specific roles of these soluble factors in TBEV infection.

## Conclusions

Immunopathological reactions in the brain make substantial contributions to the neurological sequelae during flavivirus encephalitis [56]. Considerable research efforts are now focused on targeting pro-inflammatory cytokines and chemokines as a novel and promising therapy for various diseases, including viral neuroinfections [56, 57]. For example, it may be possible to reduce immunopathological reactions in the brain by neutralizing inflammatory cytokines or antagonizing their receptor function [57]. Our present study is important because the results delineate the major inflammatory cytokines and chemokines present in the serum or brain tissue during infection in the TBE mouse model and provide a description of their production kinetics. Moreover, we identified both neuronal cells and astrocytes as one of the potential sources of these soluble factors in the infected brain. Future studies are needed to investigate the suitability of the identified cytokines/chemokines as potential targets of immunomodulatory therapy.

## Data Availability

The datasets used and/or analyzed during the current study are available from the corresponding author on reasonable request.

## Abbreviations

CSF: Cerebrospinal fluid
DMEM: Dulbecco’s Modified Eagle Medium
GFAP: Glial fibrillary acidic protein
HBCA: Human primary cortical astrocytes
IFN: Interferon
IL: Interleukin
ISG: Interferon-stimulated gene
m.o.i.: Multiplicity of infection
PS: Porcine kidney stable
TBE: Tick-borne encephalitis
TBEV: Tick-borne encephalitis virus
UV: Ultraviolet
